# The Development of a Measurement Tool Evaluating Knowledge Related to Sensory Processing among Graduate Occupational Therapy Students: A Process Description

**DOI:** 10.1155/2017/6713012

**Published:** 2017-01-11

**Authors:** Bryan M. Gee, Kelly Thompson, Jane Strickland, Lucy Jane Miller

**Affiliations:** ^1^Idaho State University, Pocatello, ID, USA; ^2^University of Colorado, Denver, CO, USA

## Abstract

With an increased demand arising from stake holders to provide more complex clinical experiences and to have students better prepared for clinical rotations, educators need to develop instructional tasks and measures to teach and assess clinical reasoning. The purpose of this article is to highlight a clinical simulation measure revolving around the A SECRET reasoning approach, which is also generalizable to other conditions and interventions. Preliminary findings of 1st year Master of Occupational Therapy students (*n* = 8) who took part in a pilot of the A SECRET case scenario reported positive, yet not strong, attitudes toward the A SECRET assessment and the sensory processing related content delivered in an online format as a part of a larger study. Overall the student perceptions and the processes of the measure development suggest an inherent value of using the proposed type of simulated case scenarios in assisting occupational therapy students in their program's first year with the development of clinical reasoning.

## 1. Introduction

Occupational therapy (OT) educators train entry level OT students to identify, evaluate, and treat children with sensory processing difficulties and disorders [1]. According to the* 2011 Accreditation Council for Occupational Therapy Education (ACOTE) Standards and Interpretive Guide *and the American Occupational Therapy Association's (AOTA)* Blue Print for the Future of Entry Level Education* [2], entry level occupational therapists should demonstrate competency in assessing clients' sensory needs and providing stimulation and environmental self-management strategies to individuals with sensory processing deficits [3].

It has been reported that 83–90% of occupational therapists working with pediatric populations provide interventions to address their clients' sensory processing deficits [4]. Green et al. [5] reported that a sensory processing approach was the third most requested intervention strategy by parent's/caregiver's children with ASD.

There are also different types of comprehensive intervention approaches being implemented by occupational therapists, Ayres Sensory Integration Intervention® [6] and the Sensory Treatment and Research Center Sensory Processing Approach [7]. In addition, there are several sensory processing resources available to occupational therapists to guide them in their interventions. However, the clinical reasoning or decision-making process used by therapists to select a frame of reference or intervention has not been widely reported in the literature.

The OT profession has placed an increased emphasis on the generation of evidence supporting the efficacy of different types of practices and interventions used by therapists to increase occupational participation and performance in our clients [8]. Yet there is less of a focus regarding how to teach students a formalized process for determining when and how to use evidenced based interventions.

The purpose of this article is to outline the development and evaluative process of a clinical simulation measure designed to capture student knowledge and clinical reasoning following completion of an online course on sensory processing education. Additionally, it is our aim to provide readers with a blue print for developing similar clinical simulation measurement tools, regardless of the intervention topic being taught, to capture student reasoning.

## 2. Review of the Literature

Clinical reasoning has been defined as the thought process used by occupational therapists during evaluation and intervention to design, implement, and modify a therapeutic plan of care [9, 10]. Crabtree [11] characterized clinical reasoning as “the process of how therapists make sense of clinical situations and how they decide to proceed in therapy” (p. 113). Schell and Schell [9] articulated it as “the process used by practitioners to plan, direct, perform, and reflect on client care” (p. 131). Higgs and Jones [12] further stated clinical reasoning is a therapist's ability to take into consideration the needs, wishes, and ideals of the client.

Throughout the therapy process, therapists have to draw conclusions and act on their assumptions about the client and the interventions that are available to make decisions without the advantage of sustained reflection [9, 13]. A central element of clinical reasoning in occupational therapy is critical thinking [14]. Critical thinking has been defined as “reflective thinking focused on deciding what to believe or do” [15, p. 1]. There are several models of critical thinking [15, 16] in which the therapist proceeds through a process of steps to recognize a problem, attempt to understand it, analyze it, evaluate it through a diverse lens, and generate possible solutions [17]. Critical thinking is a necessary ability to engage in clinical reasoning in occupational therapy practice [14]. There are numerous tools available to measure students' ability to think critically. However, there are few objective tools available to educators to measure a student's ability to clinically reason.

In entry level occupational therapy education, there are few* formative* tools to measure a student's ability to clinically reason. The profession relies on several summative mechanisms including the National Board Certification in Occupational Therapy examination [18], the Occupational Therapy Knowledge Evaluation [18], completion of a curriculum course of study and institutional graduation, and successful completion of 24 weeks of Level II fieldwork education in which a fieldwork educator subjectively rates a student's ability to evaluate a client's occupational needs, select appropriate assessment tools to identify causes contributing to a client's occupational performance problems, design safe and effective interventions, and evaluate the outcomes of therapy [3]. These measurement tools determine whether a student is able to use the knowledge garnered during the education process. Educators continue to seek formative tools that will allow them to identify problems in students' clinical reasoning within the academic setting in order to remediate issues prior to working with “real live clients.”

McCarron and Amico [19] examined the impact of problem based learning (PBL) on students' clinical reasoning using case studies. Student performance was evaluated after the PBL in a foundational occupational therapy course. The researchers subjectively evaluated the students' clinical reasoning within their written responses for four case studies for accuracy and reflection. Coker [20] used the Self-Assessment of Clinical Reflection and Reasoning [21] and California Critical Thinking Skills Tests [22] to evaluate the clinical reasoning and critical thinking of occupational therapy students engaged in a week long constraint induced movement intervention camp for children with hemiparesis. Using a case study evaluation approach [23] evaluated 1st year MOT students clinical reasoning abilities by analyzing student journal entries as they responded to predetermined questions that were rooted in different types of clinical reasoning (e.g., ethical, procedural, and narrative reasoning).

Faculty among some medical schools have used the Script Concordance approach to assess the clinical reasoning of medical students [24]. The reasoning measure requires students to generate a clinical hypothesis using objective diagnostic data, then the instructor adds additional information such as a clinical sign, lab report, and finally the student makes a diagnostic decision based on the previous information provided. Students then are graded based on the accuracy of their clinical hypothesis and diagnostic decision [24].

There is evidence of use of e-learning instructional delivery to facilitate or enhance existing clinical reasoning among OT students during fulltime clinical rotations after the completion of didactic course work [14, 17, 25–28]. Scanlan and Hancock [14] explored online synchronous and asynchronous interaction of students completing clinical rotations and reported that the participants demonstrated an increase with the quality of their clinical reasoning (procedural reasoning), increased understanding of the diagnostic presentation of clients (procedural or scientific reasoning), increased problem solving within client cases, and greater implementation of OT practice models as a part of the evaluation and intervention processes.

Poulton et al. [29] explored the value of virtual patient cases with premedical students. The authors examined students' perception of having what they termed as “multiroute virtual cases,” which allowed the students to make different decisions and experience the consequence. This type of problem based learning was implemented in opposition to “linear paper cases” which included one path to a possible outcome. The accuracy of the student's decisions was determined by expert review and opinion, with objective data being generated related to the students decisions. It was reported that 75% of the students (*n* = 72) preferred to use the multiroute virtual cases over the traditional approaches. The medical tutors who assisted the students indicated that the premedical students were more engaged when interacting with the virtual multiroute. The researchers attributed this to the experience of exploring different decisions and their consequences.

Overall there are gaps within the literature regarding the process by which occupational therapy students learn to clinically reason within didactic instructional methods, specifically, how occupational therapy students learn to reason through clinical problems based upon intervention approaches for specific conditions.

## 3. Method

As a part of a larger study which created an online, self-paced module related to sensory processing interventions, Gee [30] developed an objective measurement tool assessing how occupational therapy students reasoned through challenging behaviors rooted in sensory processing. Following the self-paced online module, 1st year Master of Occupational Therapy students were given an online examination in which they had to view a case study (written client history and online video clips of the client demonstrating challenging behaviors related to sensory overresponsivity). After viewing this material, the students were asked to complete the clinical reasoning measure by rank ordering a list of strategies from most inappropriate to most appropriate for each of the seven intervention elements of the A SECRET model. Finally, students were required to provide a rationale as to why they rank ordered the various strategies the way they did.

### 3.1. Explanation of the Measure

The primary objective of the measure was to determine if first year OT students could use the A SECRET (Attention, Sensation, Emotion Regulation, Culture, Relationships Environment, and Task) reasoning approach to accurately discriminate among varying levels (exemplary to poor) of intervention strategies. The measure was also designed to require students to justify their intervention strategies choices. This additional requirement allowed the researcher to examine thematic elements that emerged within their clinical reasoning process for the justification of their choices.

**Figure 1:**
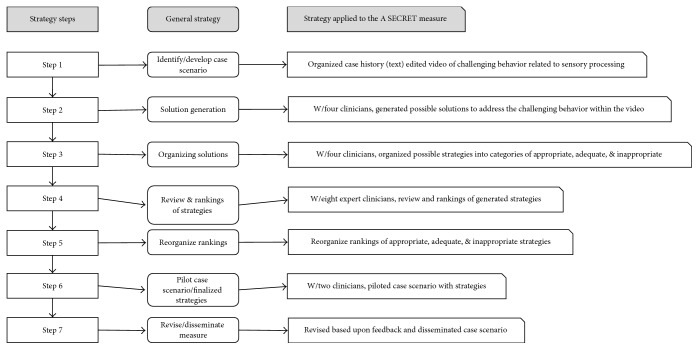
Case scenario development process.

The original developer of the A SECRET reasoning approach did not go beyond identifying the specific elements of A SECRET (Attention, Sensation, Emotion Regulation, and so on). The A SECRET reasoning approach was developed to provide parents, teachers, and other caregivers with tools to help them start thinking more like occupational therapists [7, 31] when addressing challenging behaviors related to sensory processing. The process has been disseminated in print [31] and through face to face instruction [32]. The primary author attempted to create a measure that would evaluate a student's adherence to the A SECRET principles and the student's ability to discriminate between A SECRET strategies that are appropriate and inappropriate based on a case study. Please refer to Figure 1 to see a visual representation of the process used to develop the measure.

### 3.2. Measurement Development Process

**Step 1 .**

The process of designing and developing the measure began with isolating a case study/vignette that overtly exemplified a challenging behavior related to sensory processing disorders, specifically sensory modulation disorder: overresponsivity. In this case, a 5-year-old child who was diagnosed with and Autism Spectrum Disorder and sensory overresponsivity to tactile and auditory sensations was used. A case history was established based on clinical observations, standardized testing, and parental report/concerns. This information was combined with 5 minutes of video footage that captured the child having difficulty participating in a school holiday program due to sensory related behaviors. The case history and video vignette were combined and presented as the case scenario in which the A SECRET reasoning process would then be based on.

**Step 2 .**

After developing the case scenario, a list of six or more strategies for each element of A SECRET was generated by two third year MOT students and two pediatric community occupational therapists from the Pocatello, Idaho area. All of the participants had experience using the A SECRET problem solving approach while on clinical rotation or as a part of their routine practice.

**Step 3 .**

The strategies that were generated in Step 2 were edited for clarity and grammar and reviewed by the researcher to ensure that each strategy was at least plausible and aligned with the developmental history document and the video vignette within the case scenario. Six strategies for each of the seven elements were finalized and incorporated into a document for later review and expert rankings.

**Step 4 .**

Further validation of the strategies for each element was conducted using eight experienced occupational therapists employed at the Sensory Treatment and Research (STAR) center in Denver, Colorado. For a description of the participants from the STAR Center, please refer to Table 1 for a summary of the rater demographics.

After reviewing the case history and the video vignette each rater ranked the strategies for each element from 1 to 6, with 6 being the most inappropriate strategy and 1 the most appropriate strategy. In addition to providing their ratings, the raters were asked to provide rationales for the two strategies they had ranked as 1 and 2 (appropriate) and for the two considered to be inappropriate (ranked as 5 and 6). Ranking an item a 4 or 5 meant that the strategy was considered neutral.

**Step 5 .**

The primary researcher organized the compilation of ratings for the appropriate and inappropriate strategies and subsequent adequate ratings. Strategies attaining 75% or higher agreement for consensus among the raters were then deemed appropriate or inappropriate and ranked as such in the measure. When the ratings did not meet the desired 75% threshold, the expert opinion of the developer of the A SECRET reasoning process was obtained to establish the final rating(s). The rank order was then adjusted based upon their final ratings of the strategies. Refer to Table 2 an example of the expert ratings.

**Step 6 .**

After the strategies were reorganized based on the feedback from the therapist's ratings from the Sensory Treatment and Research (STAR) center (Denver, Colorado), the completed measure was uploaded into a web-based learning management system and was piloted with two pediatric occupational therapists in the Pocatello, Idaho area. Feedback was gathered regarding the delivery of the measure, the measure's instructions, the overall process of the measure (rankings of strategies and strategy rationale), issues related to the user interface, and navigation within the learning management system.

**Step 7 .**

Final revisions were completed based on feedback from the piloted review. The measure was then administered to eight 1st year MOT students as a part of a larger study exploring the effectiveness of A SECRET e-learning modules.

### 3.3. Evaluation of the Measure

As a part of the analysis of the measure, reliability of the A SECRET elements was calculated using the sample of 1st year MOT students in order to determine the internal consistency using Cronbach's alpha, *α* [33]. Cronbach's alpha for the entire A SECRET assessment was .61, which demonstrated low to moderate internal consistency. However, for each construct (i.e., element) there was poor to moderate internal consistency. The Attention category on the assessment (questions 1–6) was .67, which is moderate; the Sensation category (questions 7–12) was −.30, which is very poor; the Emotion Regulation category (questions 13–18) was .36, which is poor; the Culture category (questions 19–24) was .56, which is poor; the Relationships category (questions 25–30) could not be calculated due to the fact that all participants were 100% accurate in their ratings; the Environment category (questions 31–36) was −.81, which is very poor; and the Task category (questions 37–42) was .67, which is moderate. The questions with low internal validity will be revised and reassessed for further analysis.

The processes of the A SECRET case scenario measurement development have been presented to enable replication and refinement related to additional measures of the A SECRET reasoning approach as well as for the development of measures of clinical reasoning using differing approaches to reasoning or interventions and diverse populations. It is hoped that interested educators and clinicians will use the outlined process for the creation and use of other measurement resources for the development of clinical reasoning among occupational therapy students and practitioners. It was apparent that there was difficulty for experienced clinicians to come to consensus on the “appropriate strategy rankings” which then challenges students to demonstrate the capacity to make informed clinical decisions related to sensory processing. However, this difficulty may also be grounded in the issue that the occupational therapy does not function under manualized approaches as a part of standard care practices.

## 4. Student Feedback on the Measure

At the completion of the study, students were surveyed regarding their attitudes towards the measure. The survey used a four-point scale of strongly disagree, disagree, agree, and strongly agree. Overall, the attitudes of the participants were favorable. Descriptive statistics were collected related to the students' attitudes toward the measure's directions (M = 3.25; SD = 0.462), their perception of the face validity of the measure (M = 3; SD = 0.755), and general preference for the use of a case scenario to demonstrate their knowledge and understanding of sensory processing strategies (M = 3; SD = 0.755).

The descriptive statistics of attitudes toward the content of the A SECRET case scenario were M = 3 and Mdn = 3 with a SD = 0.755. The participants' ratings were positive but not strong. There were several factors that may have contributed to this finding. First, this may have been a novel testing experience for this cohort of students as it was exclusively online and asynchronous. The type of assessment likely was unique to their academic experience because they were asked to discriminate between six distractors and formulate a rationale to justify their ratings. Additionally, students were not informed of their performance until after they had completed a face to face focus group. However, this is a current limitation of the measure; if the measure could provide students with immediate feedback regarding the strategy they selected, the measure would become a true instructional tool that not only measures clinical reasoning but is able to educate and improve a student's clinical reasoning skills.

## 5. Discussion

There was a positive perception among the participants regarding the case scenario. Yet, their attitudes may have been influenced by the novelty of the assessment and the fact that they were asked to provide input toward using an assessment measure. The participants' perceptions could have been higher had two specific factors been addressed prior to administration of the measure. The researcher purposely inserted time restrictions that forced the participants to complete the entire assessment in 60 minutes and limited the number of views of the simulated client's developmental history and video vignette. These decisions were based on the researcher's desire to create a “real life” effect, by attempting to replicate some aspects of clinical practice. The participant's wanted to have more time to complete the measure and the option to return to the initial case video and history for further review. In routine clinical practice the OT may have only one chance to observe a challenging behavior that is disrupting performance; the OT does not have the luxury to refer back to a video or other information. Instead, she/he must rely on memory and judgments made as they were initially accessing the information. Hence, these factors were foundational to ensure that the experience was more than merely a selected-response test and that they are a reflection of clinical reasoning within a simulated occupational therapy session.

In another study, Williams et al. [34] study reported occupational therapy students who participated in an interprofessional education (IPE) DVD-based simulation; the occupational therapy students highly valued the simulation process of the IPE DVD. Our study's findings support the findings of Williams et al. [34] as the 1st year MOT students found the simulation portion of the assessment valuable.

Interestingly, the profession of occupational therapy has begun exploring different types of simulation within entry level OT education as recently as 2014 [35] via an instructional practice pattern survey. Additional research has been conducted on the mechanics of simulation and possible implications for occupational therapy education and clinical practice [36]. Yet, at the time of the development of the measure and pilot study, the literature was sparse related to outcomes and perceptions of simulation among consumers of occupational therapy education. Additionally, the studies that were explored primarily focused on the subjective analysis of clinical reasoning among students. These studies lacked objective parameters to assess the effectiveness of a student's clinical reasoning based on an occupational performance problem and the implementation of an intervention. The findings and descriptions of the participants' attitudes toward the A SECRET case scenario, though narrow, provide a starting point to address the gap in the profession's understanding regarding student perceptions of the use of simulation as part of OT education and training.

## 6. Implications for Occupational Therapy Education

There is an increased demand on the time and resources of occupational therapy educators towards scholarship and service. This is confounded with the expectation from fieldwork educators that more students arrive for Level II fieldwork rotations to hit the ground running. The present study is timely and provides a possible process to objectively assess clinical reasoning of students before they embark on structured clinical fieldwork rotations. This article also presents student feedback to this type of assessment and is an indicator of the value students place on this type of assessment of their knowledge and performance.

## Figures and Tables

**Table 1:** Raters demographics.

Year graduated	Degree	Years working with pediatric populations	Years using A SECRET
1974	Research doctorate	40	17
1981	Research doctorate	32	10
1991	Master degree	22	10
1999	Clinical doctorate	7	7
2001	Bachelor degree	13	10
2007	Master degree	6	6
2010	Master degree	3	3
2011	Master degree	2	2

**Table 2:** Expert ratings for strategies listed for the Task element in A SECRET.

Task										
Rater 1	Rater 2	Rater 3	Rater 4	Rater 5	Rater 6	Rater 7	Rater 8	Total	Strategy	Combined
6	6	6	6	6	6	6	6	8/8 = I	Remove Michael from the music program to sit in the audience.	Inappropriate strategy average = 71%
4	3	5	1	5	2	4	5	3/8 = I	Have Michael focus less on singing and more on the fine motor movements/gestures.	
5	2	4	5	4	1	5	4	3/8 = N	Have Michael focus less on the fine motor movements/gestures and more on singing the words of the songs.	
3	1	3	4	3	3	3	1	5/8 = N	Assign Michael simple jobs to help the music leader during the entire program.	

2	4	2	3	2	4	2	2	5/8 = A	Assign Michael simple physical tasks/jobs during or in between songs.	Appropriate strategy average = 57%
1	5	1	2	1	5	1	3	5/8 = A	Have the teacher/music leader include planned movements in the song/music.	

I = rating for the inappropriate strategy; N = rating for a strategy deemed neutral; A = rating for the appropriate strategy.
